# DNA methylome and transcriptome identified Key genes and pathways involved in Speckled Eggshell formation in aged laying hens

**DOI:** 10.1186/s12864-022-09100-8

**Published:** 2023-01-19

**Authors:** Xue Cheng, Xinghua Li, Yuchen Liu, Ying Ma, Ruiqi Zhang, Yalan Zhang, Cuidie Fan, Lujiang Qu, Zhonghua Ning

**Affiliations:** 1 National Engineering Laboratory for Animal Breeding, College of Animal Science and Technology, China Agricultural University, Beijing, 100193 China; 2 Rongde Breeding Company Limited, Hebei, 053000 China

**Keywords:** Laying hens, Speckled eggs, Transcriptome, DNA methylation, Immunity

## Abstract

**Background:**

The quality of poultry eggshells is closely related to the profitability of egg production. Eggshell speckles reflect an important quality trait that influences egg appearance and customer preference. However, the mechanism of speckle formation remains poorly understood. In this study, we systematically compared serum immune and antioxidant indices of hens laying speckled and normal eggs. Transcriptome and methylome analyses were used to elucidate the mechanism of eggshell speckle formation.

**Results:**

The results showed that seven differentially expressed genes (DEGs) were identified between the normal and speckle groups. Gene set enrichment analysis (GSEA) revealed that the expressed genes were mainly enriched in the calcium signaling pathway, focal adhesion, and MAPK signaling pathway. Additionally, 282 differentially methylated genes (DMGs) were detected, of which 15 genes were associated with aging, including *ARNTL*, *CAV1*, and *GCLC*. Pathway analysis showed that the DMGs were associated with T cell-mediated immunity, response to oxidative stress, and cellular response to DNA damage stimulus. Integrative analysis of transcriptome and DNA methylation data identified *BFSP2* as the only overlapping gene, which was expressed at low levels and hypomethylated in the speckle group.

**Conclusions:**

Overall, these results indicate that aging- and immune-related genes and pathways play a crucial role in the formation of speckled eggshells, providing useful information for improving eggshell quality.

**Supplementary Information:**

The online version contains supplementary material available at 10.1186/s12864-022-09100-8.

## Background

Poultry eggs are one of the most important protein sources, and their relatively low cost makes them popular among consumers. Over the last four decades, egg production has improved considerably due to the development of specialized egg breeds and genetic selection, with the target of “Feeding laying hens to 100 weeks to produce 500 eggs” [1, 2]. However, achieving this target has been limited by the gradual decline in eggshell quality and physiology associated with hen aging, resulting in increased eggshell weight, lighter eggshell color, and speckled eggshells [3].

The reddish-brown speckle, an important eggshell quality trait, often appears on the blunt end of the brown eggshell, considerably affecting egg appearance and customer preference. The degree of eggshell speckling is evaluated using a scoring method. Speckles can be scored according to pigment intensity, distribution, and speckle size [4]. Moreover, the heritability of speckled eggshells ranges from 0.15–0.2, indicating genetic determination [5]. Additionally, a previous study indicated that aged hens have a higher rate of speckled eggshells than younger hens, reaching 20% after 60 weeks of age [6].

The eggshell gland is an egg formation organ that plays a critical role in eggshell structure and color formation. During egg formation, the yolk travels through the infundibulum, magnum, and isthmus and reaches the eggshell gland, which secretes a large amount of calcium, pigment, cuticle, and other substances, forming a complete eggshell structure and an outer cuticle [7–9]. Any modification or damage to the eggshell gland affects eggshell structure formation and pigmentation [10–12].

DNA methylation is one of the earliest known modification pathways, and involves the transfer of methyl groups to the fifth carbon site of cytosine to form 5-methylcytosine [13]. DNA methylation plays an important role in the aging process of animals, regulating age-related gene expression and the etiologies of neurological, immunological, and metabolic diseases [14–17]. Additionally, DNA methylation combined with environment factors can cause different phenotypes during aging [18]. Several complex livestock phenotypes have been linked to DNA methylation [19, 20]. Recently, RNA sequencing (RNA-seq) has been useful in revealing the genes and pathways underlying traits at the transcriptional level [21–24], such as embryonic muscle development, feed efficiency, and litter size.

Our previous study showed that, although the occurrence of speckled eggshells does not affect the performance of laying hens (unpublished data), speckles can affect the appearance of eggs and reduce their economic value considerably. Moreover, the molecular mechanisms of speckled egg formation are poorly understood. Therefore, this study aimed to elucidate the mechanism of speckled eggshell formation using transcriptomic and DNA methylation techniques. Since speckled eggshells are heritable and age-related, we used a combination of transcriptomic and DNA methylation analyses to explore the key genes and pathways involved in speckled eggshell formation. It is anticipated that the findings of this study will improve the understanding of the molecular mechanism of eggshell trait formation, which would be useful for animal breeding.

## Results

### Serum biochemical parameters

**Fig. 1:**
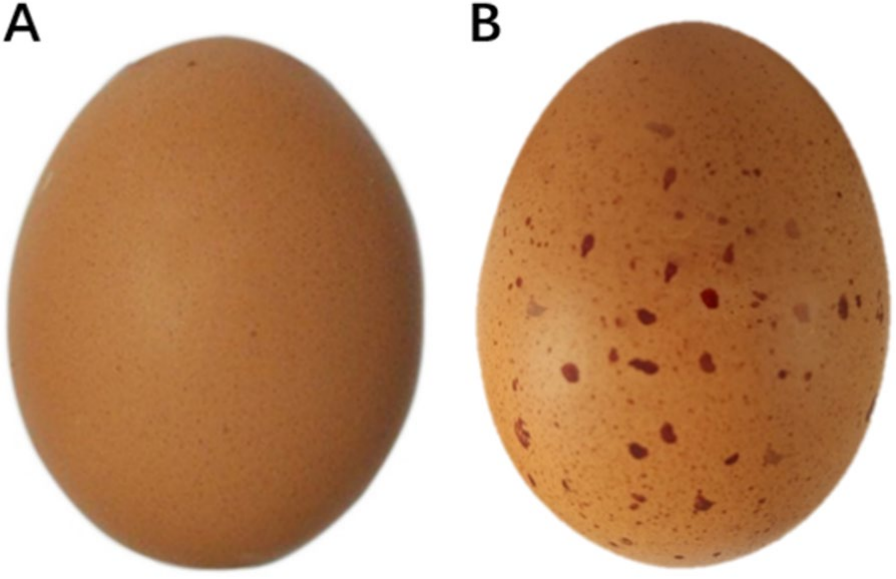
Normal and speckled eggs from aged laying hens. **A** Normal egg, **B** Speckled egg

A typical egg and speckled egg are shown in Fig. 1. Serum antioxidant and immune indices were measured to determine the physiological status of the laying hens. Serum biochemical parameters are listed in Table 1. Serum levels of immunoglobulin G (IgG) and immunoglobulin A (IgA) are common indicators of humoral immune function. Birds in the normal group had a higher (*p* = 0.028) IgA content than those in the speckle group. Superoxide dismutase (SOD), catalase (CAT), total antioxidant capacity (T-AOC), glutathione (GSH), and glutathione peroxidase (GSH-PX) are important antioxidant enzymes in the body. MDA is one of the products formed by the reaction of lipids with oxygen radicals, and its content represents the degree of lipid peroxidation. These indices are important in evaluating the oxidative stress process. However, there were no differences (*p* > 0.05) in antioxidant parameters between the normal and speckle groups.

**Table 1:** Serum biochemical parameters of the normal and speckle groups

**Parameters**	**Normal hens**	**Speckle hens**	*p*-value
IgG (g/L)	4.21 ± 0.18	4.20 ± 0.14	0.900
IgA (g/L)	2.31 ± 0.09	2.21 ± 0.07	0.028
SOD(U/mL)	154.06 ± 3.54	153.83 ± 4.11	0.905
MDA(nmol/mL)	4.93 ± 0.40	4.92 ± 0.39	0.947
GSH-PX(U/mL)	910.07 ± 64.13	911.26 ± 35.13	0.964
GSH(U/mL)	2.76 ± 0.57	2.37 ± 0.19	0.098
CAT(U/mL)	11.70 ± 1.05	11.58 ± 0.54	0.780
T-AOC(U/mL)	10.53 ± 1.04	10.37 ± 0.57	0.719
LZM(U/mL)	166.84 ± 9.30	165.94 ± 5.96	0.820

*CAT* catalase, *LZM* lysozyme, *MDA* malondialdehyde, *GSH* glutathione, *GSH-PX* glutathione peroxidase, *SOD* superoxide dismutase, *T-AOC* total antioxidant capacity

### Transcriptome profile of the eggshell gland

**Fig. 2:**
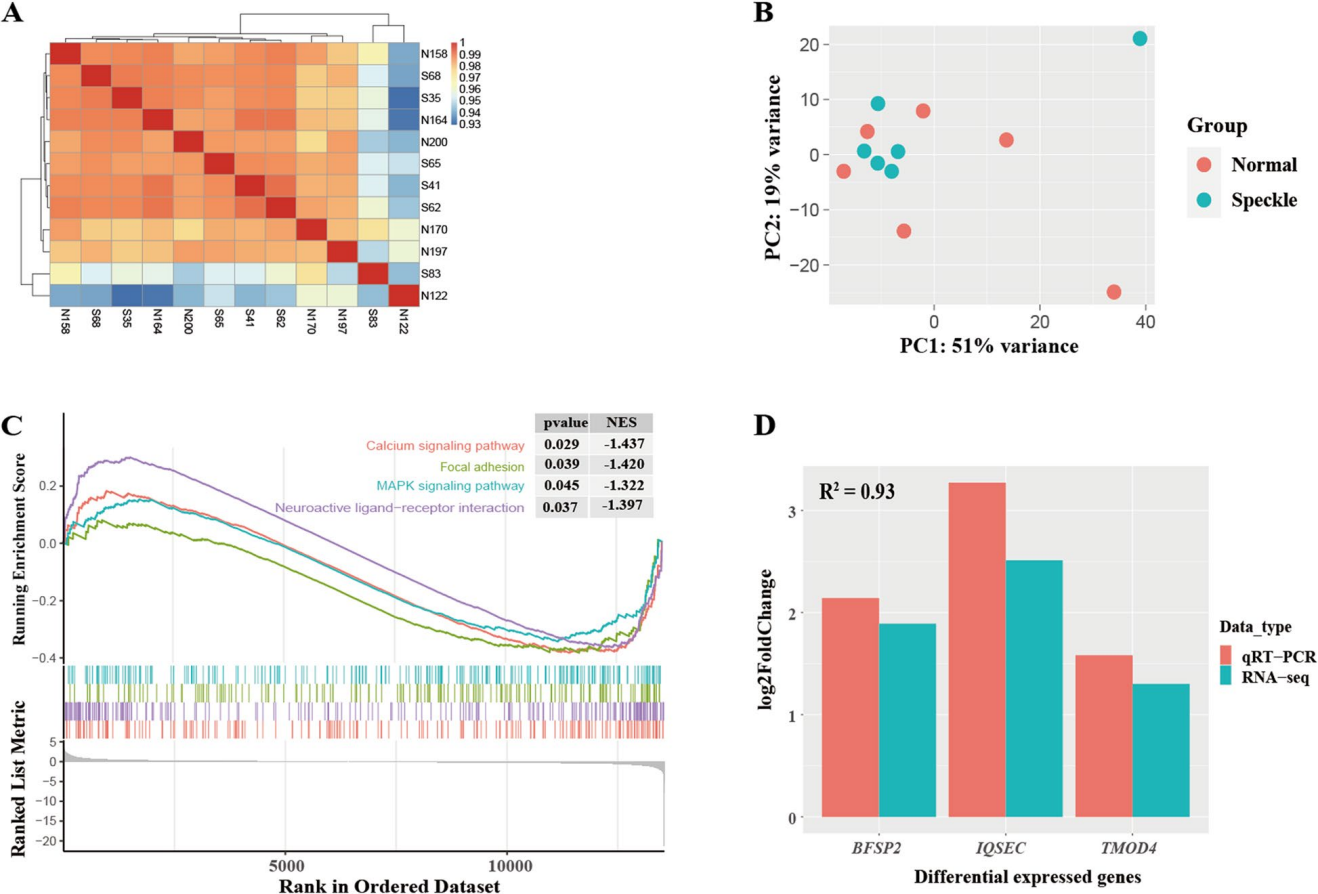
The transcriptome profile of the eggshell gland. **A** Heatmap of gene expression levels, **B** Principal component analysis of all genes using DEseq2 normalized expression values, **C** Three representative gene sets from the gene set enrichment analysis results, **D** The qPCR verification results of differentially expressed genes

Six cDNA libraries were constructed from the speckle and normal groups. After quality control, a total of 615,170,158 raw reads and 604,275,600 clean reads were obtained (98.22% of the raw reads). After alignment using HISAT2 software, the mapping rate was 90.75–93.18%, and the unique mapping rate in all samples was greater than 73.47% (Supplementary Table S3). Gene expression levels are illustrated using a cluster heatmap and principal component analysis (PCA). There were no significant differences in the gene expression profiles of samples from the speckle and normal groups, as the samples did not form distinct clusters (Fig. 2A, B). A total of seven differentially expressed genes (DEGs) were identified between the normal and speckle groups (*p* < 0.05, |log_2_ Fold Change|> 1), including two upregulated and five downregulated genes (Table 2).

**Table 2:** Differentially expressed genes (DEGs) between the hens that laid speckle and normal eggs

Ensemble ID	Gene	log_2_ Fold Change	P-adj
ENSGALG00000012975	*IQSEC3*	2.518181	3.25E-06
ENSGALG00000034585	*BFSP2*	1.890066	1.61E-05
ENSGALG00000030247	*TMOD4*	1.29841	0.007295
ENSGALG00000047412	*LOC112530987*	1.250791	0.025581
ENSGALG00000043143	*Pseudogene*	1.233585	0.021603
ENSGALG00000053989	*TRIQK*	-1.37944	0.015271
ENSGALG00000014206	*GABRA2*	-4.79624	0.030088

*P-adj* adjusted *p* value

Gene set enrichment analysis (GSEA) showed that four pathways were significantly enriched by the expressed genes (Supplementary Table S4). Negative normalized enrichment scores (NES) indicated lower expression levels for some pathways in the normal group compared with those of the speckle group, with the calcium signaling pathway, neuroactive ligand-receptor interaction, focal adhesion, and MAPK signaling pathway being the pathways with the lowest expression (Fig. 2C).

Three DEGs (*BFSP2, IQSEC, TMOD4*) identified by RNA-seq were verified using quantitative real-time PCR (qRT-PCR). A similar expression of the three genes was evident using RNA-Seq and qRT-PCR, and the coefficient of determination (R^2^) reached 0.93 (Fig. 2D), indicating that the RNA-seq data were reliable.

### DNA methylation profile of the eggshell gland

**Fig. 3:**
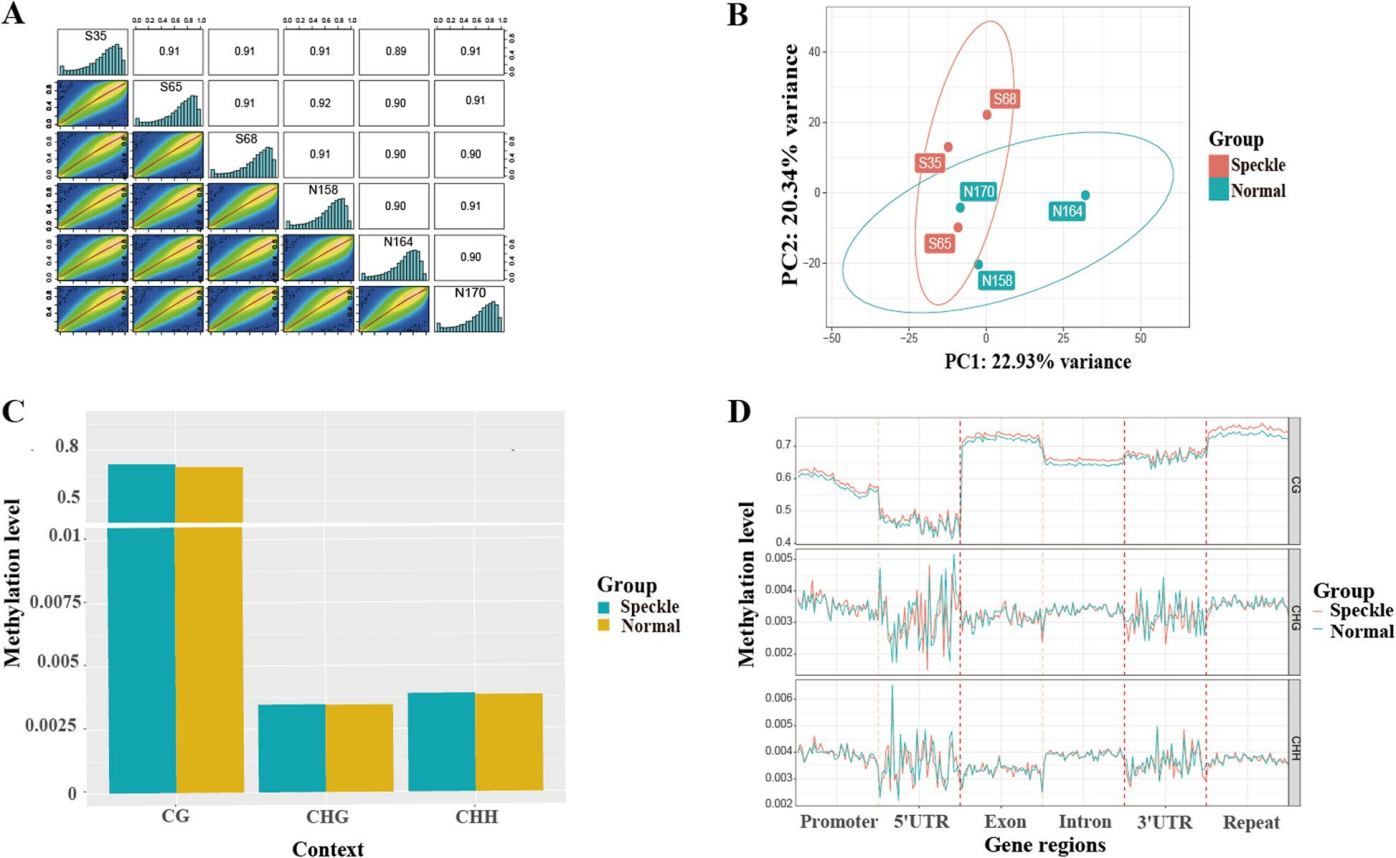
The overall methylation levels in the hens laying speckle and normal eggs. **A** Correlation analysis of methylation levels between samples from the two groups. **B** Principal component analysis of the methylation level of all samples. **C** Histogram of cytosine site methylation level in the two groups. **D** Line chart of the methylation levels of different genomic regions. The genomic regions of each gene were divided into 20 bins; the cytosine site methylation level of the corresponding functional regions of all genes was then averaged

A total of 564,415,302 and 581,414,308 clean reads were obtained from the speckle and normal groups, respectively, after quality control (Supplementary Table S5), of which 73–78% were uniquely mapped to the converted chicken reference genome (GRCg6a). The cytosine (C) methylation rate of the six eggshell gland samples was approximately 3.4%, and the cytosine site methylation of CpG ranged from 55.5–63.9% in the two groups. The cytosine site methylation of CHH and CHG (H represents A, C, or T) was detected at a low proportion (0.3–0.4%) (Supplementary Table S5). Pearson correlation analysis of the CpG bases suggested that all samples were highly correlated (*r* > 0.89) (Fig. 3A). PCA showed that samples from the two groups were not significantly different, as they did not form separate clusters (Fig. 3B). There were no significant differences in the methylation levels of CG, CHG, and CHH between the two groups (Fig. 3C). However, the speckle group showed a higher CG methylation level than the normal group. The repeat and exon regions exhibited the highest CG methylation levels, whereas the 5′ UTR region had the lowest CG methylation levels (Fig. 3D).

**Fig. 4:**
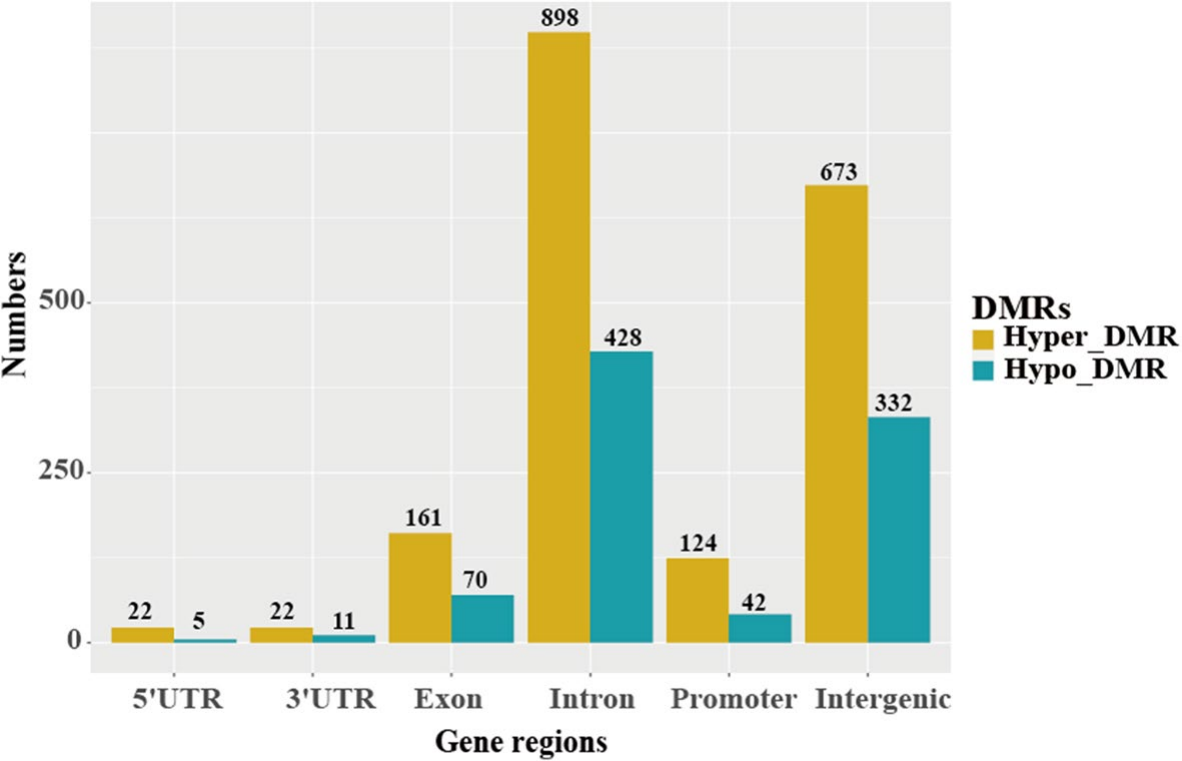
Histogram of annotation of differentially methylated regions (DMRs) in genomic functional regions

**Fig. 5:**
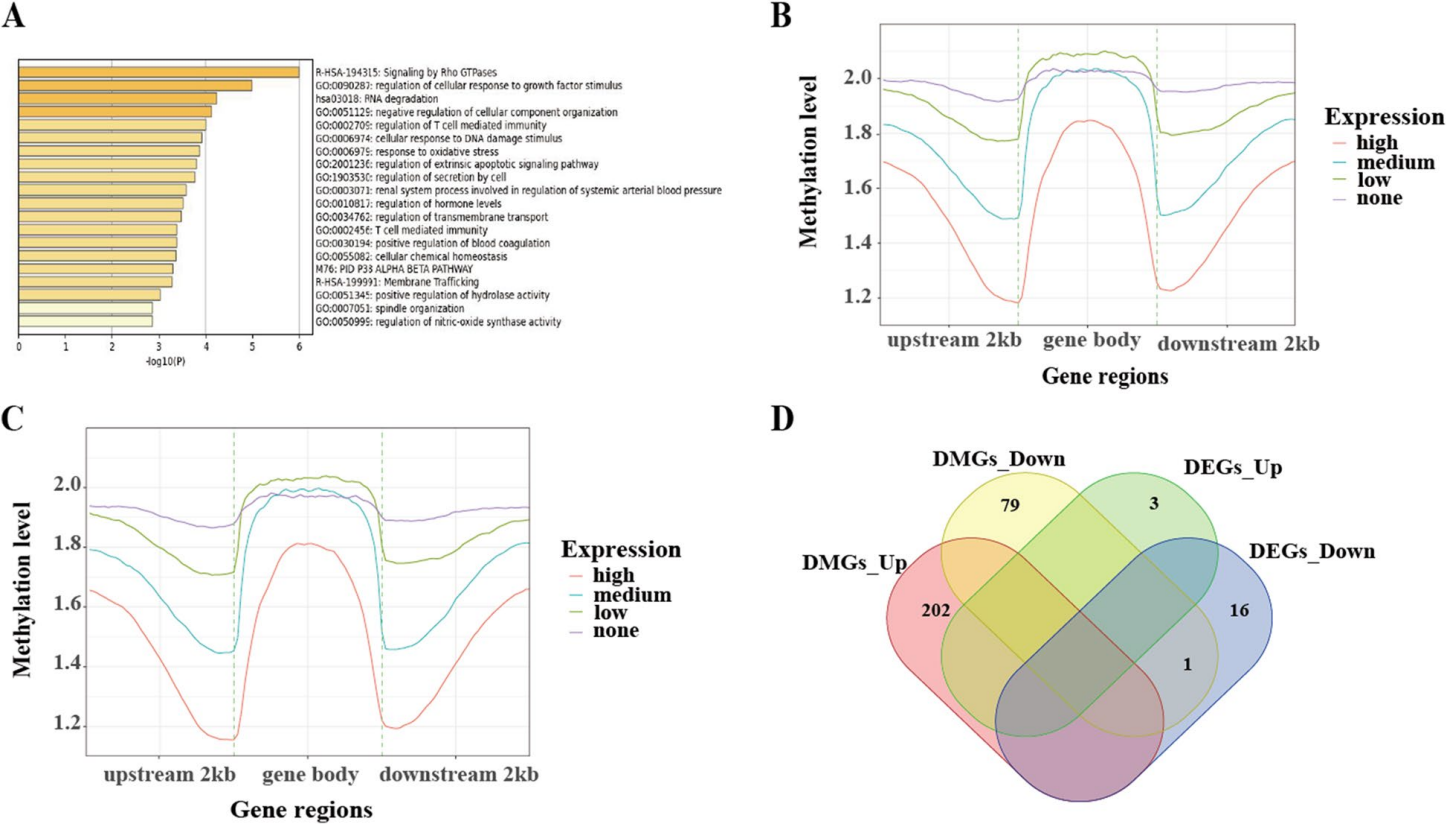
**A** GO terms of DMGs. **B** The relationship between gene expression and DNA methylation levels in the speckle group. **C** The relationship between gene expression and DNA methylation levels in normal group. **D** Venn diagram of overlapping genes between DEGs and DMGs

A total of 2788 differentially methylated regions (DMRs) were identified between the normal and speckle groups. The DMRs were mainly located in the introns (47.45%), followed by the intergenic region (36.05%), exon (8.29%), promoter (5.95%), 3′-UTR (1.18%), and 5′-UTR (0.97%) regions (Fig. 4). Additionally, 282 differentially methylated genes (DMGs) were identified, including 172 hypermethylated and 74 hypomethylated genes in the promoter region. Moreover, 36 DMGs were found in the gene body, including 30 hypermethylated genes and six hypomethylated genes. We converted DMGs into their human orthologs and obtained 158 gene symbols that were uploaded in Metascape for functional annotation, gene ontology (GO), and pathway analyses. The genes were enriched in 176 GO biological processes, including regulation of cellular response to growth factor stimulus and T cell-mediated immunity, response to oxidative stress, and cellular response to DNA damage stimulus. Pathway analysis showed that the genes were enriched in 19 Kyoto Encyclopedia of Genes and Genomes (KEGG) pathways, including RNA degradation, inflammatory mediator regulation of TRP channels, and the TNF signaling pathway. Moreover, 25 gene sets were detected, including signaling by Rho GTPases, the RHO GTPase cycle, and the CDC42 GTPase cycle. The top 20 enriched ontology clusters are shown in Fig. 5A. Among the 158 homologous human genes, 15 were related to aging or longevity (Supplementary Table S6).

An integrative analysis of the whole-genome bisulfite sequencing (WGBS) and RNA-seq data was performed to determine the relationship between DNA methylation and gene expression levels (Fig. 5B and C). There was a negative correlation between DNA methylation and gene expression levels upstream of the transcription start site (TSS) and downstream of the transcription termination site (TTS); however, there was no correlation between DNA methylation and gene expression levels in the gene body. The Venn diagram showed that *BFSP2* was the only overlapping gene between DMGs and DEGs (Fig. 5D).

## Discussion

Studies over several years have shown that the formation of speckles on eggshells is heritable, and aged laying hens produce a higher rate of speckled eggshells than younger hens [6]. Aging of laying hens is often accompanied by chronic inflammation and oxidative stress [25, 26]. The blood parameters of animals reflect their physiological and nutritional status. SOD, GSH-PX, GSH, CAT, and T-AOC are components of the antioxidant defense system. The results of the present study indicate that there was no significant difference in the antioxidant capacity of aged hens between the speckle and normal groups, which is contrary to the findings of Moreno and Osorno [27]. Moreno reported that birds laying eggs with speckled shells may suffer physiological stress because of the pro-oxidant function of the main speckle component and have a higher tolerance of oxidative stress [28]. The differences in the results could be attributed to the different species used in the studies.

Immunoglobulins are proteins involved in anti-inflammatory processes and play regulatory roles in inflammatory reactions [29]. A previous study showed that Blue Tit birds laying speckled eggs exhibit low total immunoglobulin levels [30]. Similarly, the results of the present study showed that birds in the speckle group had substantially lower serum IgA levels compared with those in the normal group, indicating that hens in the speckle group may have a lower anti-inflammatory ability, which is consistent with the findings of Martínez and Merino [30]. IgA, as one of the important indicators in evaluating the humoral immune function of poultry, has antiviral, antibacterial, and antitoxin functions [31]. Studies have shown that dietary supplementation of yeast β-glucan can increase the serum IgA content. The increased IgA content can increase an individual's ability to maintain immune homeostasis, leading to possible health benefits [32]. Studies have also shown that as laying hens age, their resistance to external pathogens decreases [33]. When pathogens enter the body of aged laying hens, the mucosal defense system of oviduct tissues may be damaged due to the concomitant decrease in immunity [34]. However, a higher level of IgA may increase the body's ability to maintain immune homeostasis, preventing the invasion of pathogens into submucosal tissue of the oviduct, thus maintaining good eggshell quality.

Furthermore, RNA-seq analysis identified seven DEGs between the speckle and normal groups, including *IQSEC3*, *BFSP2*, *TMOD4*, *LOC112530987*, *GABRA2*, *TRIQK*, and a pseudogene. *IQSEC3*, a member of the brefeldin A-resistant ARF guanine nucleotide exchange factors (GEFs) family [35, 36], promotes the development of inhibitory synapses by binding with gephyrin [37]. *IQSEC3* is functionally important for maintaining network activity in vivo. *IQSEC3* knockdown in the hippocampal dentate gyrus in rodents decreases the density of GABAergic synapses and increases susceptibility to severe seizures [38]. However, there are no studies on the function of *IQSEC3* in poultry.

Gamma-aminobutyric acid (GABA) is the main inhibitory neurotransmitter in the central nervous system of vertebrates [39], and can change the structure of the receptor and the ion permeability of the corresponding receptor [40]. GABA can act directly on tubular smooth muscle cells through GABAA-R or GABAB-R, which are located on the fallopian tube wall and participate by regulating tubular contractility in rabbits [39], humans [41], and rats [42]. Studies have shown that GABRA1 plays an important role in egg production. High expression levels of GABRA1 can inhibit the proliferation of granulosa cells, enhance cell apoptosis, and inhibit the synthesis and secretion of progesterone, resulting in reduced egg production [43–45]. The egg-laying rhythm of laying hens is a neuromodulatory process [46, 47]. Based on these results, we speculate that there is a subtle relationship between eggshell speckle formation and the nervous system, although the precise link requires further investigation.

TMOD4*,* a member of a family of proteins that cap the pointed ends of actin filaments [48], is expressed in skeletal muscle and the heart [49, 50]. Studies have reported that *TMOD4* is present in adult chicken lenses, erythrocytes, and fast twitch skeletal muscle fibers [51]. TMOD4 has been found to participate in myofibril assembly, muscle contraction, and differentiation [52–54]. The contraction of oviduct muscle makes the egg rotate in the uterus, allowing pigment to be evenly deposited on the eggshell surface. We speculate that the formation of speckled eggs may be related to oviduct muscle contraction, but further research is needed.

GSEA was used to analyze the biological function of all expressed genes to avoid the loss of some interesting gene sets through the cutoff-free strategy. The expressed genes were mainly enriched in the calcium signaling pathway, focal adhesion, the MAPK signaling pathway, and neuroactive ligand–receptor interaction. It has been reported that in the process of eggshell mineralization, genes related to the calcium signaling pathway are involved in the absorption of calcium and carbonate ions from the blood and are transported to the uterine fluid via the epithelial cells of the oviduct to participate in eggshell mineralization [46, 55]. When the calcium and carbonate ions continue to mineralize in the eggshell, the egg rotates continuously in the uterus, and the eggshell pigment, protoporphyrin-IX, can be uniformly deposited on the eggshell surface [56]. We speculate that the difference in the supply of carbonate and calcium ions between the two groups might have led to the uneven distribution of pigment in the deposition process on the eggshell surface, causing eggshell speckles.

Focal adhesions are macromolecular structures that form mechanical connections between the intracellular actin cytoskeleton and extracellular matrix components [57]. It has been shown that focal adhesions play a vital role in maintaining the morphology and function of the oviduct in Chinese brown frogs [58], while focal adhesions have also been involved in the mechanism of egg production differences in Jinghai Yellow chickens and Nandan-Yao domestic chickens [59, 60]. In the current study, focal adhesions were involved in the formation of eggshell speckles, but the specific participatory mechanism still requires further investigation.

The MAPK pathway has three prominent members: p38 mitogen activated protein kinase (p38 MAPK), Jun N-terminal kinase (JNK), and extracellular response kinase (ERK), which together regulate cell growth, differentiation, apoptosis, inflammation, and other important physiological responses [61]. The ERK 1/2 MAPK pathway has been demonstrated to play an important role in the growth, development, and differentiation of the oviduct and uterus [62]. Wang et al. used Vanadium to induce the growth of oviduct epithelial cells, and found that MAPK family members were activated, leading to oxidative stress of the oviduct, reduced cell activity, and cell apoptosis [63]. In the current study, we found that the MAPK signal pathway in the speckled group was substantially enriched, and therefore speculate that the hens laying speckled eggs might have experienced some stress, with some impact on the oviduct. Neuroactive ligand–receptor interactions are related to steroid hormone synthesis in the gonads. They play an essential role in the regulation of egg production and ovarian function in poultry [59, 64]. The differentially expressed gene, *GABRA2*, also belongs to the neuroactive ligand–receptor interaction pathway. Since eggshell speckles appear as a result of egg production, and egg production is a rhythmic process, the substantial enrichment of neuroactive ligand–receptor interactions in the speckled group leads us to speculate that there may be differences in egg production performance between hens laying speckled eggs and hens laying normal eggs, but this requires further exploration.

Environmental factors can affect gene expression via epigenetic modifications. The combination of genetic and epigenetic modifications could be helpful in explaining the formation mechanism of complex traits [19, 65]. The relationship between genome-wide DNA methylation and gene expression has been studied for years [66, 67]. Generally, DNA methylation represses gene expression [68]. The results of the present study are consistent with previous findings in that high gene expression levels are associated with low DNA methylation in the promoter region [67, 68]. However, no obvious trend was observed in the gene body region, which may be because gene expression patterns are also regulated by other factors [69, 70]. Functional enrichment analysis revealed that DMGs were mainly enriched in T cell-mediated immunity, response to oxidative stress, and cellular response to DNA damage stimuli, and most of the pathways are associated with aging [71–73]. Other aging-related genes have also been identified, including *GCLC* [74], *CAV1* [75], and *LYN* [76].

A combination of transcriptome and DNA methylation data showed that *BFSP2* was the only overlapping gene and was significantly expressed at both DNA methylation and transcriptomic levels. *BFSP2* was hypomethylated and its expression levels were low in the speckle group, suggesting gene expression is regulated by other transcription factors in addition to methylation modification. Moreover, *BFSP2* has been identified as a candidate gene in autosomal-dominant congenital cataracts [77] and progressive cataract disease [78]. Although it has been shown that *BFSP2* is related to eye development in chickens [79], studies on other functions in chickens are limited. Therefore, further studies are necessary to elucidate its role in speckled eggshell formation.

## Conclusions

In conclusion, the serum immune indices indicate that the IgA content in the speckled group was substantially lower than that in the normal group, and we speculate that a decreased immune function is closely correlated with eggshell speckle formation. Transcriptome analysis detected seven DEGs between the speckle and normal groups, of which *IQSEC3*, *GABRA2*, and *BFSP2* were identified as potentially important genes associated with speckled egg formation. DNA methylation analysis identified DMGs associated with T cell-mediated immunity, response to oxidative stress, and cellular response to DNA damage stimulus. Of the 282 DMGs identified, 15 were associated with aging. Integrative analysis of transcriptome and DNA methylation revealed that the only overlapping gene was *BFSP2*, which has barely been studied in chickens. The data presented here suggest that the immune and aging pathways of laying hens may contribute to speckled eggshell formation, improving our understanding of the mechanism of generation of eggshell speckles.

## Methods

### Ethical statement

All chicken were obtained from Rongde Breeding Company Ltd. (Hebei, China). Ethical and animal welfare issues were approved by the Ethics Committee of China Agricultural University (permit number: AW02602202-1–1). All experimental protocols were performed according to the Ministry of Science and Technology (Beijing, China). We declare that this study is reported in accordance with ARRIVE guidelines.

### Animals

A total of 2000 Island Red hens (60-weeks old) were raised in the same environment in individual cages at Hebei Rongde Poultry Breeding Co. Ltd., Hebei, China. The appearance of the eggs was observed according to the method of Cheng [6] to identify individuals that consistently laid speckled and normal eggs, and those laying heavily speckled eggs and normal eggs; hens were divided into two groups (8 hens per group): speckle and normal groups. The hens had free access to drinking water and were fed at a fixed time every day in an enclosed chicken house under a standard lighting program of 16:8 h (light:dark). The temperature in the chicken house was maintained at 18 ± 1 °C and the relative humidity at 55 ± 5%. All hens were free of avian leukosis viruses and *Salmonella pullorum*.

### Eggshell gland collection

Eggshell speckles are mainly located in the cuticle and vertical crystal layer (unpublished data). Eggshell mineralization stops and the cuticle is deposited in the final 1.5 h before egg expulsion into the eggshell gland [9, 56]. Considering the gene expression dynamics during different stages of egg production, each chicken was sampled at approximately 1.5 h before laying by observing and recording the laying time of the two groups for 7 d. On d 8, eggshell glandular mucosa was collected during the eggshell pigment formation period, placed in cryopreservation tubes, and quickly immersed in liquid nitrogen for preservation.

### Serum biochemical parameters

Blood samples were collected from the wing vein of laying hens in the normal (*n* = 8) and speckle (*n* = 8) groups into coagulant collection tubes, placed at room temperature for 3 h, and then centrifuged for 10 min at a rate of 3,500 rpm. Serum and plasma were collected for the analysis of blood immune and antioxidant indices. IgG and IgA were used to evaluate the immune performance of an individual hen. SOD, GSH-PX, GSH, CAT, and T-AOC are antioxidant enzymes, while MDA is the product of oxidative stress and is usually used together with antioxidant enzymes as a key indicator to measure antioxidant capacity. Lysozyme (LZM) is used to measure an individual's ability to clear pathogens. IgG, IgA, SOD, MDA, GSH-PX, GSH, CAT, T-AOC, and LZM levels were measured using the colorimetric method with a spectrophotometer (UV-1750, Shimadsu, Japan). The commercial kits of IgG (E026-1–1), IgA (E027-1–1), SOD (A001-1–2), MDA (A003-1–2), GSH-PX (A005-1–2,), GSH (A006-1–1), CAT (A007-1–1), T-AOC (A015-1–2), and LZM (A050-1–1) were purchased from Nanjing Jiancheng Bioengineering Institute (Nanjing, China).

### RNA and DNA extraction, library preparation, and sequencing

DNA samples (*n* = 3 per group, Supplementary Table S1) and total RNA samples (*n* = 6 per group, Supplementary Table S1) were extracted from the eggshell gland tissue using the animal genomic DNA kit (DP304, Tiangen, Beijing, China) and Trizol reagent (Cat. No. 155–96-026, Invitrogen Life Technologies, Carlsbad, USA), according to the manufacturer’s instructions. DNA and RNA quality and concentration were measured using a spectrophotometer (NanoDrop Technologies, Rockland, DE, USA). Qualified DNA and RNA samples were sent to Personalbio (Shanghai, China) and Majorbio (Shanghai, China), respectively, for library construction. Transcriptome and whole-genome bisulfite sequencing were performed using the Illumina HiSeq X Ten (Illumina, San Diego, CA, United States).

### RNA-seq data analysis

Raw reads were quality assessed and filtered using fastp software (0.20.1) [80]. Clean reads were aligned to the chicken reference genome (GRCg6a) using HISAT2 (2.2.1) [81]. The sequencing alignment/mapping (SAM) file was converted to a binary alignment/mapping (BAM) file and sorted using SAMtools (1.11) [82]. The number of reads mapped to each gene was calculated using featureCounts [83]. DEGs were identified using DEseq2 based on the following criteria: P-adjust < 0.05 and |log_2_ Fold Change|> 1 [84]. GSEA was used to identify significantly different genes in the two groups [85]. Compared with the functional enrichment analysis of DEGs, GSEA uses a cut-free strategy and accumulates subtle expression changes in each gene to compare biological differences. All expressed genes were ranked according to the fold-change value between the two groups. The enrichment score and NES were calculated for each gene set, and significantly different gene sets were identified based on |NES|> 1 and a *p*-value < 0.05.

### Quantitative real-time PCR

Total RNA was extracted from 12 eggshell gland tissues (six hens laying normal eggs and six hens laying speckled eggs) using an RNA extraction kit (Tiangen, Beijing, China) following the manufacturer’s specifications. The mRNA was reversely transcribed into cDNA using a PrimeScript™ RT reagent kit with gDNA Eraser (Takara, Dalian, China). Three DEGs (Due to insufficient samples, we only verified three DEGs, *BFSP2, IQSEC, TMOD4*) and *GAPDH* were selected to design primers based on the chicken coding sequence from the NCBI database (Supplementary Table S2). *GAPDH* was selected as the internal control. The qRT-PCR was conducted using the TB Green® Premix Ex Taq™ Kit (Takara, Kusatsu, Japan) with an ABI 7500 system (Applied Biosystems), using the following program: 95 °C for 30 s, 40 cycles of 95 °C for 5 s, 55 °C for 30 s, 72 °C for 30 s. The 2^−ΔΔCt^ method was used to calculate the relative expression level. The R^2^ between qRT-PCR and RNA-seq was calculated using simple linear regression in R Studio.

### WGBS data analysis

Sequence quality, adapter filtering, and low-quality reads were screened using Fastp (0.20.1) [80]. The clean data were fully bisulfite-converted, aligned against the bisulfite-converted reference sequence, and de-duplicated using Bismark software (0.22.3) [86]. A bismark methylation extractor was used to determine methylation status. The methylation levels of CpG, CHH, and CHG were calculated using the sliding-window approach [87]. The MethylKit package was employed to identify DMRs and DMGs [88]. DMRs were defined when the differential methylation level was > 20% at *p* ≤ 0.05. Genes that overlapped with the DMRs were defined as DMGs. The region from the TSS to the TTS was defined as the gene body region. The region 2 kb upstream of the TSS and 2 kb downstream of TTS was defined as the promoter region. Pathway enrichment analysis was conducted using Metascape (https://metascape.org/) with default parameters to annotate the functions of DMGs [89].

### Integrative analysis of transcriptome and WGBS Data

To obtain the expression profile of DNA methylation and transcriptome levels, all genes were divided into four groups according to their expression levels, and the average methylation level was calculated based on each group. Overlapping genes between the DMGs and DEGs were analyzed using a Venn plot.

### Statistical analysis

EXCEL and SPSS 25.0 (SPSS Inc., Chicago, IL, USA) were used for statistical analysis. Student’s *t*-test was used to compare the two groups at a significance level of 5% (*p* < 0.05). The results are presented as the mean ± standard error.

## Supplementary Information

**Additional file 1: Supplementary Table 1.** Information on sequencing samples.

**Additional file 2: Supplementary Table 2.** Specific primers for qRT-PCR.

**Additional file 3: Supplementary Table 3.** Summary statistics for transcriptome sequence quality and alignment information of eggshell gland.

**Additional file 4: Supplementary Table 4.**  The gene set between between normal and speckle group.

**Additional file 5: Supplementary Table 5.** Summary statistics for WGBS alignment and methylation information of eggshell gland.

**Additional file 6: Supplementary Table 6.** Genes related to senescence and longevity.

## Data Availability

The RNA sequencing raw data are available at: https://dataview.ncbi.nlm.nih.gov/object/PRJNA850950?reviewer=u9h3k9jracbqg9fkjeabjl2sm2. The WGBS raw data are available at: https://dataview.ncbi.nlm.nih.gov/object/PRJNA851109?reviewer=k9miop7kfajh6l2mcoa6jha6pq.

## Abbreviations

DEG: Differentially expressed gene
DMG: Differentially methylated gene
RNA-seq: RNA sequencing
WGBS: Whole-genome bisulfite sequencing
DMR: Differentially methylated region
TSS: Transcriptional start site
TTS: Transcriptional terminal site
PCA: Principal component analysis
GO: Gene ontogeny
KEGG: Kyoto Encyclopedia of Genes and Genomes
