# Supplementary material for: DNA methylome and transcriptome identified Key genes and pathways involved in Speckled Eggshell formation in aged laying hens
Source: BMC Genomics. 2023 Jan 19;24:31. doi: 10.1186/s12864-022-09100-8 (PMC9854222; doi:10.1186/s12864-022-09100-8)
Supplement: Supplementary file 6 — Additional file 6: Supplementary Table 6. Genes related to senescence and longevity. [file 12864_2022_9100_MOESM6_ESM.docx]

Supplementary Table 6. Genes related to senescence and longevity.

| Gene Symbol |
| --- |
| ARHGAP1[1], ARNTL [2], ATRIP [3], CAV1 [4], ERCC6 [5], FAM49B [6], GCLC [7], LY86 [8], SORCS2[8], LYN [9], LYZ [10], MGST1[10], SIK1[11], TRPM8[12], VPS8[13] |

**Reference:**

1. Wang L, Yang L, Debidda M, Witte D, Zheng Y: **Cdc42 GTPase-activating protein deficiency promotes genomic instability and premature aging-like phenotypes**. *Proc Natl Acad Sci U S A* 2007, **104**(4):1248-1253.

2. Kondratov RV, Kondratova AA, Gorbacheva VY, Vykhovanets OV, Antoch MP: **Early aging and age-related pathologies in mice deficient in BMAL1, the core componentof the circadian clock**. *Genes Dev* 2006, **20**(14):1868-1873.

3. Yashin AI, Wu D, Arbeev KG, Ukraintseva SV: **Polygenic effects of common single-nucleotide polymorphisms on life span: when association meets causality**. *Rejuvenation Res* 2012, **15**(4):381-394.

4. Park DS, Cohen AW, Frank PG, Razani B, Lee H, Williams TM, Chandra M, Shirani J, De Souza AP, Tang B *et al*: **Caveolin-1 null (-/-) mice show dramatic reductions in life span**. *Biochemistry* 2003, **42**(51):15124-15131.

5. van der Pluijm I, Garinis GA, Brandt RM, Gorgels TG, Wijnhoven SW, Diderich KE, de Wit J, Mitchell JR, van Oostrom C, Beems R *et al*: **Impaired genome maintenance suppresses the growth hormone--insulin-like growth factor 1 axis in mice with Cockayne syndrome**. *PLoS Biol* 2007, **5**(1):e2.

6. Murgia M, Toniolo L, Nagaraj N, Ciciliot S, Vindigni V, Schiaffino S, Reggiani C, Mann M: **Single Muscle Fiber Proteomics Reveals Fiber-Type-Specific Features of Human Muscle Aging**. *Cell Rep* 2017, **19**(11):2396-2409.

7. Orr WC, Radyuk SN, Prabhudesai L, Toroser D, Benes JJ, Luchak JM, Mockett RJ, Rebrin I, Hubbard JG, Sohal RS: **Overexpression of glutamate-cysteine ligase extends life span in Drosophila melanogaster**. *J Biol Chem* 2005, **280**(45):37331-37338.

8. Sebastiani P, Solovieff N, Dewan AT, Walsh KM, Puca A, Hartley SW, Melista E, Andersen S, Dworkis DA, Wilk JB *et al*: **Genetic signatures of exceptional longevity in humans**. *PLoS One* 2012, **7**(1):e29848.

9. Park JW, Ji YI, Choi YH, Kang MY, Jung E, Cho SY, Cho HY, Kang BK, Joung YS, Kim DH *et al*: **Candidate gene polymorphisms for diabetes mellitus, cardiovascular disease and cancer are associated with longevity in Koreans**. *Exp Mol Med* 2009, **41**(11):772-781.

10. de Magalhaes JP, Curado J, Church GM: **Meta-analysis of age-related gene expression profiles identifies common signatures of aging**. *Bioinformatics* 2009, **25**(7):875-881.

11. Ferrand M, Kirsh O, Griveau A, Vindrieux D, Martin N, Defossez PA, Bernard D: **Screening of a kinase library reveals novel pro-senescence kinases and their common NF-kappaB-dependent transcriptional program**. *Aging (Albany NY)* 2015, **7**(11):986-1003.

12. Yee NS, Brown RD, Lee MS, Zhou W, Jensen C, Gerke H, Yee RK: **TRPM8 ion channel is aberrantly expressed and required for preventing replicative senescence in pancreatic adenocarcinoma: potential role of TRPM8 as a biomarker and target**. *Cancer Biol Ther* 2012, **13**(8):592-599.

13. Fabrizio P, Hoon S, Shamalnasab M, Galbani A, Wei M, Giaever G, Nislow C, Longo VD: **Genome-wide screen in Saccharomyces cerevisiae identifies vacuolar protein sorting, autophagy, biosynthetic, and tRNA methylation genes involved in life span regulation**. *PLoS Genet* 2010, **6**(7):e1001024.
